# Development and practical application of condition monitoring system for ultra-high voltage electrical equipment

**DOI:** 10.1038/s41598-025-33285-z

**Published:** 2026-01-13

**Authors:** Shuxin Hu, Ming Meng

**Affiliations:** Department of Electrical Engineering, North China Electric Power University, Baoding, 071051 China

**Keywords:** Ultra-high voltage electrical equipment, Infrared monitoring, SF6 gas, Surge arrester, Condition monitoring, Energy science and technology, Engineering, Materials science

## Abstract

Electrical equipment serves as mission-critical infrastructure in power systems. Operational status monitoring is fundamental to load demand fulfillment. This study employs intelligent methodologies to establish an ultra-high voltage equipment condition monitoring system for real-time operational status surveillance, enabling timely fault diagnosis and early warning through automated analysis of equipment status changes. Key metrics: GIS SF6 gas density achieves 6.452 kg/m³ under ambient cooling conditions; SF6 gas pressure reaches 0.960 MPa during switching operations; SF6 gas temperature declines to -28.491℃ under high-frequency switching cycles; Surge arrester leakage current exhibits non-monotonic behavior with voltage escalation, with peak magnitude observed at 95.591 mA; Under full load, the GIS three-phase voltage reaches 239.958 V. The three-phase current exhibits an overall downward trend, dropping to 2.519 A under heavy load. Active power peaks at 3,472.504 W under medium load, while reactive power reaches 1,078.828 var under heavy load. Apparent power attains 4,431.743 VA under heavy load. The power factor initially decreases, then rises, achieving 0.904 at full load. The successful development of this system provides valuable insights for enhancing equipment reliability, improving monitoring efficiency, and reducing operational costs.

**Supplementary Information:**

The online version contains supplementary material available at 10.1038/s41598-025-33285-z.

## Introduction

The 13th WTO Ministerial Conference called for addressing both current and emerging regulatory challenges, particularly in areas like sustainable development and the digital economy. The G20 leaders’ Antalya Summit highlighted the importance of taking action in the energy sector. These efforts are crucial in tackling climate change and its impacts. As urban electricity consumption continues to rise, the development of ultra-high voltage systems highlights the need for smarter and more efficient operation of equipment^1^. Electrical equipment is a crucial part of the power system, and monitoring its operational status is essential for ensuring a reliable supply of electricity to meet demand^2^. However, their failure risk is increasing due to prolonged operation under high loads and in complex environments^3^. Ultra-high voltage electrical equipment is large-scale and complex, with numerous internal components operating in a constantly changing state. State monitoring systems for such equipment provide real-time insights into its operating conditions with minimal disassembly, allowing for early detection of faults and prompt resolution of issues.

Sun-Jae Kim et al. eliminated the need for specialized cables and wires by adopting wireless technology, achieving wireless temperature monitoring^4^. Rathnayaka, Sooriya Bandara et al. employed online impedance extraction technology based on the inductive coupling method to monitor early signs of electrical equipment defects^5^. R. Ahmed and his team developed a new UHF antenna design for the continuous and efficient detection of partial discharge in medium-voltage gas-insulated applications^6^. Hu Zishu and his team implemented online monitoring of dissolved gases in the insulating oil of electrical equipment and validated the data, providing a solid foundation for real-time analysis of the equipment’s operational condition^7^. Zhang Fengyi applied machine learning algorithms and AI technologies to create precise models for analyzing real-time data, achieving fault prediction and diagnostic capabilities^8^. Li Xingsong and his team leveraged big data technology to analyze vast volumes of equipment operation data, significantly improving the real-time capabilities and accuracy of monitoring^9^.

The research highlights that efficient and intelligent monitoring of ultra-high voltage electrical equipment is crucial for maintaining the stability of the power supply. Therefore, developing smart monitoring methods for these devices, along with precise fault diagnosis, is essential. However, the existing methods have limitations—traditional monitoring cannot provide real-time status updates and still depends on manual inspections and maintenance. To tackle the challenges outlined above, this paper uses intelligent methods to develop a state monitoring system for ultra-high voltage electrical equipment, offering a detailed analysis of how to achieve real-time monitoring of operational status and implement early warning functions.

## The principles of monitoring ultra-high voltage electrical equipment

The online monitoring of power equipment is primarily carried out through the use of electronic engineering, communication, and sensor technologies. During the monitoring process, careful selection of the right monitoring parameters is crucial^10^. An analog-to-digital conversion model is established to optimize the coordination of data transmission and packet indicators across different ports, focusing on stages such as preprocessing templates, compression requirements, and the transmission of key values through communication paths^11^.

At present, there are several major approaches to monitoring the condition of electrical equipment. The first category involves monitoring through traditional methods^12^. The second category includes AI-driven methods, such as machine learning and deep learning techniques^13^. Additionally, a logical inference approach for interpretable electrical equipment faults, based on the fusion of time series data, is also employed^14^.

For ultra-high voltage electrical equipment, non-contact infrared temperature measurement technology effectively satisfies the monitoring requirements, enabling precise detection of thermal faults in the equipment^15,16^. The characteristics of infrared radiation are outlined as follows^17^.

The propagation speed of infrared radiation in a vacuum is:

1 $$C = \omega \lambda = 3 \times 10^{8} \left( {m/s} \right)$$

In the equation:

$\:\mathrm{C}$ — speed of propagation ($\:\mathrm{m}/\mathrm{s}$); $\:{\upomega\:}\:$— angular frequency ($\:\mathrm{r}\mathrm{a}\mathrm{d}/\mathrm{s}$); $\:{\uplambda\:}$ — wavelength ($\:{\upmu\:}\mathrm{m}$).

Based on Wien’s law, the peak wavelength of infrared radiation emitted from an object’s surface is:

2 $$\:{\uplambda\:}\mathrm{p}\:=\:\frac{2898}{T}\:\left({\upmu\:}\mathrm{m}\right)$$

In the equation:

$\:{\uplambda\:}\mathrm{p}$ — peak wavelength ($\:{\upmu\:}\mathrm{m}$); $\:\mathrm{T}$ — object’s absolute temperature ($\:\mathrm{K}$).

According to the Stefan-Boltzmann law, the infrared radiation power of an object is:

3 $$\:\mathrm{P}\:={\upepsilon\:}\times\:{\upsigma\:}\times\:{\mathrm{T}}^{4}(\mathrm{W}/{\mathrm{m}}^{2})$$

In the equation:

P — total radiant power emitted by an object (W);

$$\:{\upepsilon\:}$$ — emissivity;

$\:{\upsigma\:}$ — Stefan-Boltzmann constant, which equals $5.67 \times 10^{{ - 8}} W/\left( {m^{2} \cdot K^{4} } \right)$ in nature;

T — thermodynamic temperature (K).

**Fig. 1:**
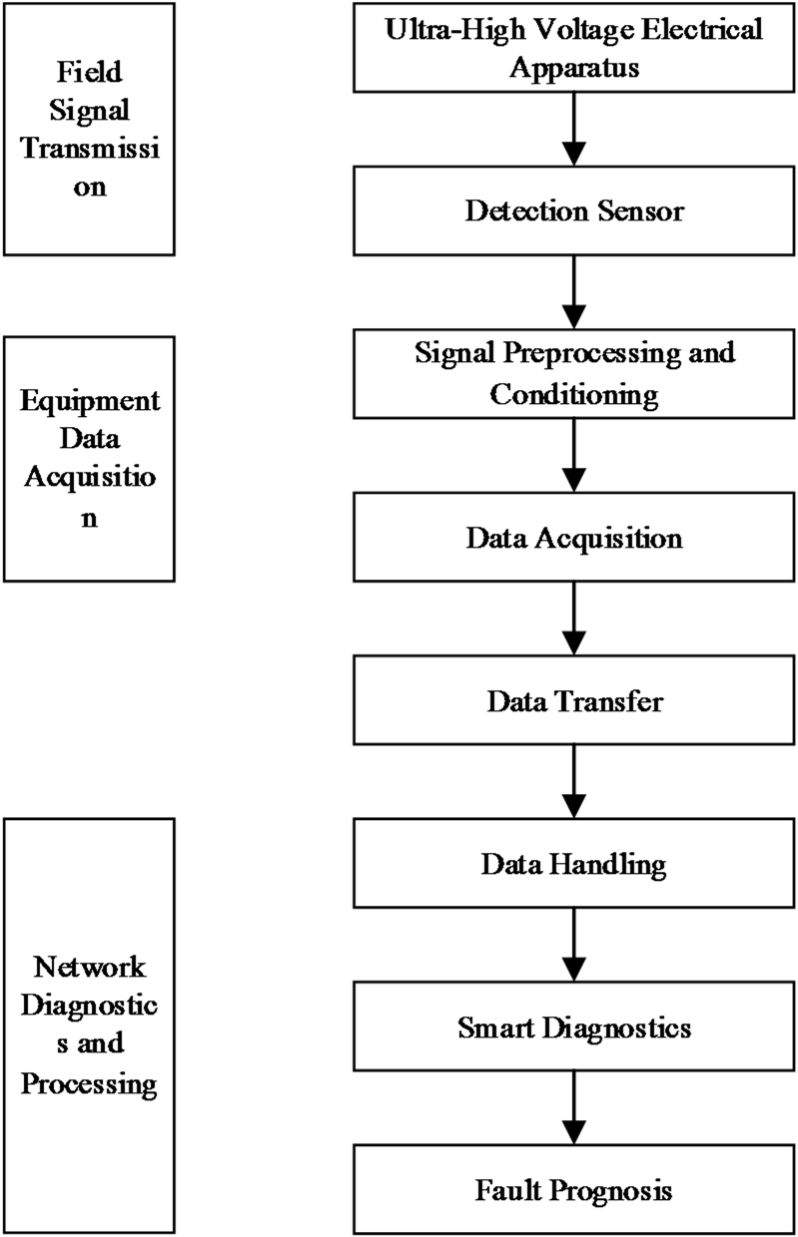
Schematic of the ultra-high voltage electrical equipment online monitoring system.

The principle of the ultra-high voltage electrical equipment online monitoring system is shown in Fig. 1.

## Test results and analysis of ultra-high voltage electrical equipment monitoring

**Fig. 2:**
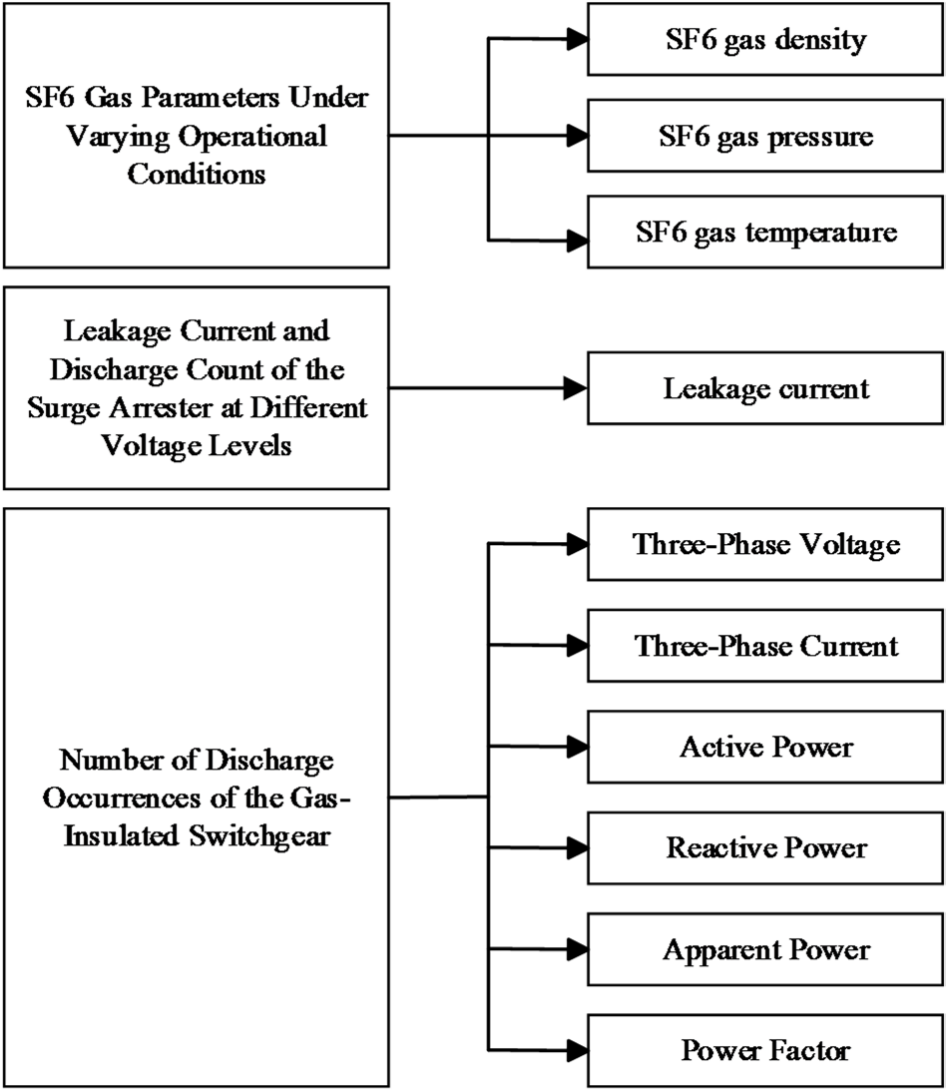
Experimental workflow diagram.

This experimental study focuses on ultra-high voltage gas-insulated switchgear (GIS), incorporating critical components such as SF6 circuit breakers and surge arresters, with the objective of parameter measurement under varied operational conditions. The setup consists of sensors, data acquisition units, transmission devices, and software, designed to capture parameters, and convert them into digital signals for real-time transmission. When the GIS parameters at the sensor installation point reach the predefined thresholds, the standalone system will trigger an alarm. All graphical data presented in this study are derived from real-time measurements, with analytical datasets being directly acquired from the monitoring instrumentation. The experimental procedure is illustrated in Fig. 2.

### SF6 gas parameters of the SF6 circuit breakers under varying operational conditions

SF6 gas density is measured using a gas density relay, with a detection range of 0 to 10.00 kg/m3 and a measurement uncertainty of ≤ 5%.

SF6 gas pressure is measured using a gas density relay, with a detection range of 0 to 1.00 MPa and a measurement uncertainty of ≤ 5%.

SF6 gas temperature is measured using a temperature sensor, with a detection range of −50 to 100 °C and a measurement uncertainty of ≤ 1 °C.

The SF₆ density relay detects gas leaks and triggers alarms or shutdowns to prevent accidents. Its performance is crucial for electrical equipment safety^18,19^. Over time, sensor drift and aging can reduce reliability, making regular calibration^20^ and testing vital for power system stability^21,22^.

Common calibration methods include SF₆ relay calibrators and temperature chamber testing^23,24^. The chamber method simulates different temperatures, ensuring accurate and reliable full-range calibration^25^.

#### SF6 gas density under varying operational conditions

**Fig. 3:**
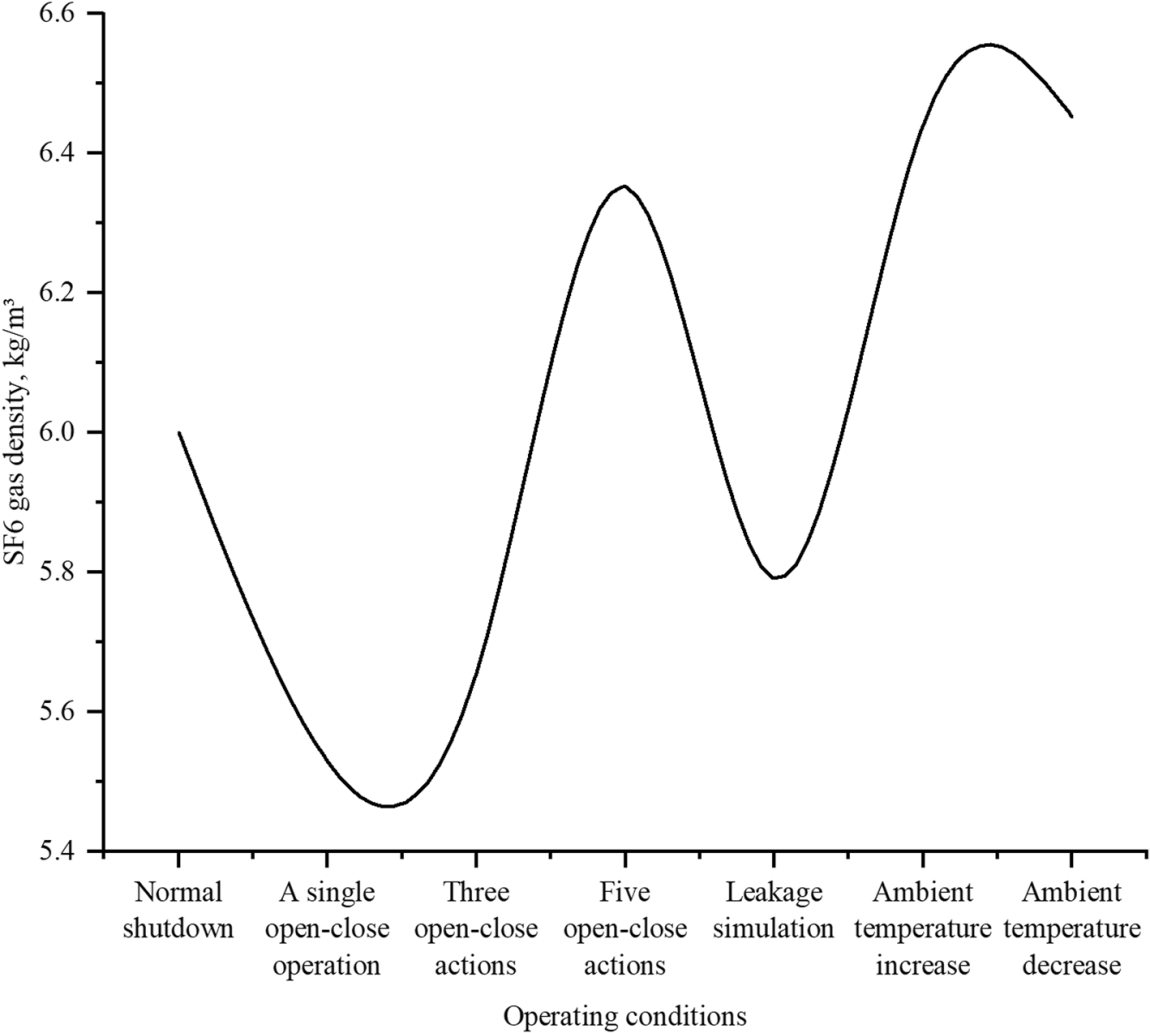
SF6 gas density.

Figure 3 shows SF6 gas density variations under different conditions, with key data points analyzed. Normal shutdown maintains stable density (6.000 kg/m³), while one open-close cycle shows brief fluctuations (5.529 kg/m³). Multiple cycles (three/five) exhibit more pronounced density changes (5.656 kg/m³, 6.351 kg/m³), potentially due to leakage or pressure variations. Simulated leakage demonstrates a clear density decline (5.791 kg/m³). Ambient temperature changes affect density (6.441 kg/m³ for increase, 6.452 kg/m³ for decrease), though system sealing and pressure may moderate this relationship. Interference conditions promote more uniform gas distribution and faster sensor response^26^. Breaker operation frequency shows no direct correlation with mechanical wear, as fewer operations may still yield higher failure rates^27^.

SF6 gas density critically influences insulation performance in GIS equipment. When the density falls below the threshold value of 5.5 kg/m³, the insulation strength deteriorates significantly, leading to an increased partial discharge activity and system frequency deviations. Experimental data demonstrate that at densities below 5.5 kg/m³, partial discharge frequency rises from 0 to 1 to 5–10 occurrences per day, with discharge magnitude increasing from tens to hundreds of pC. Correspondingly, system frequency deviations expand from ± 0.01 Hz to beyond ± 0.05 Hz, indicating compromised voltage stability.

#### SF6 gas pressure under varying operational conditions

**Fig. 4:**
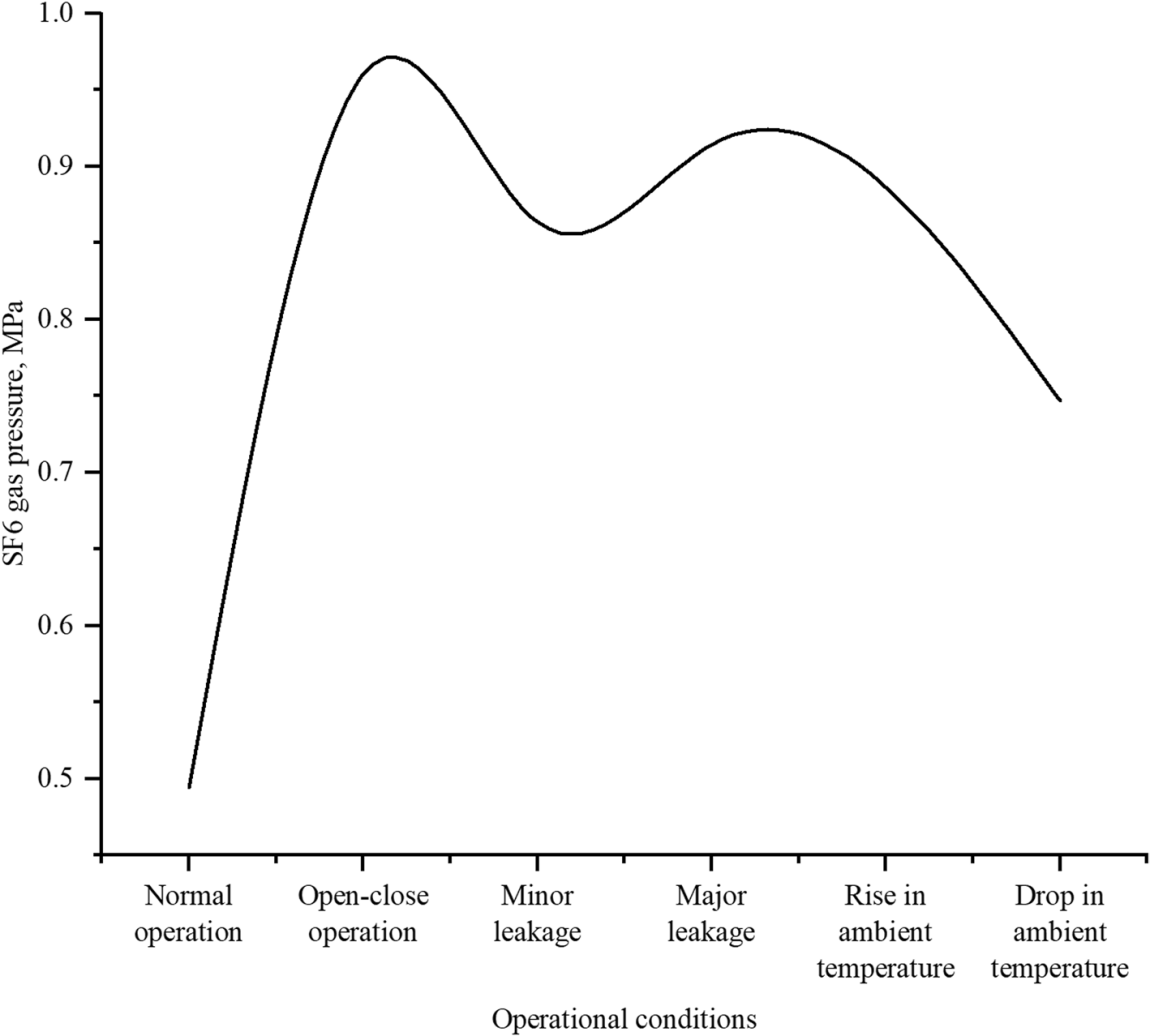
SF6 gas pressure.

Figure 4 presents SF6 gas pressure variations under different operational conditions. Key observations include: normal operation maintains stable low pressure (0.494 MPa), while open-close operations cause rapid pressure surges (peak 0.960 MPa). Leakage conditions exhibit distinct patterns - minor leakage (0.864 MPa) shows fluctuations, whereas major leakage (0.914 MPa) demonstrates initial pressure drop followed by recovery, suggesting insufficient gas replenishment at high leakage rates^28^. Temperature variations significantly influence pressure, with increases (0.886 MPa) and decreases (0.747 MPa) corresponding to gas expansion and contraction respectively. Complementary studies on SF₆/CF₄ mixtures reveal non-monotonic pressure dependence and temperature-sensitive breakdown characteristics^29^, further elucidating the complex gas behavior under varying environmental conditions.

#### SF6 gas temperature under varying operational conditions

**Fig. 5:**
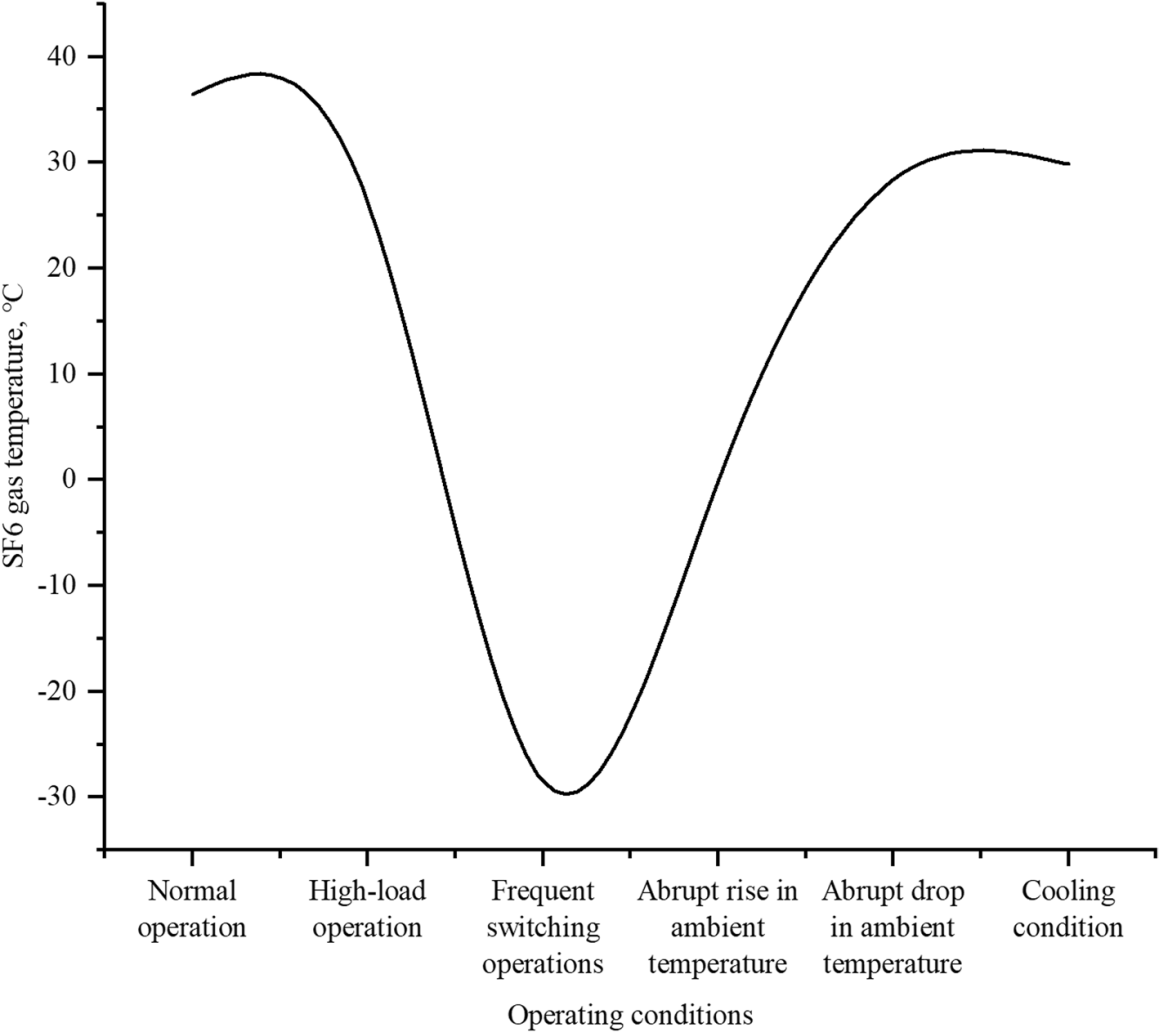
SF6 gas temperature.

Figure 5 demonstrates SF6 gas temperature variations under different operational conditions. Key observations include: normal operation maintains stable temperature (36.468 °C), while high-load operation shows transient fluctuations before stabilizing (26.265 °C). Frequent switching operations cause sharp temperature drops (−28.491 °C). Ambient temperature changes significantly influence gas temperature, with sudden increases decreasing temperature (−0.119 °C) and sudden decreases raising it (28.344 °C), indicating compensatory thermal regulation. Cooling conditions maintain stable temperatures (29.822 °C) comparable to normal operation. Notably, environmental dust affects infrared monitoring accuracy, with temperature readings stabilizing beyond a critical dust layer thickness^30^. These findings provide crucial insights into SF6 thermal behavior under varying operational and environmental stresses.

Based on the analysis of the three aspects, it can be observed that the SF6 gas parameters exhibit relatively stable variations. Over the course of one month of monitoring, data fluctuations were minimal when identical operations were performed at different times. However, the insulation performance of GIS can deteriorate gradually owing to various causes, such as minute leakage of high-pressure SF6 gas, an increase in internal particles, and the inflow of external air during high-voltage and high-current operation^31^. The trends in the variations of different SF6 gas parameters vary significantly, but all reach peak values during frequent switching operations. Additionally, all parameters show strong fluctuations in response to sudden changes in ambient temperature. In GIS installations, SF6 gas may liquefy at the coldest locations. These typically occur on the enclosure surfaces, particularly where ohmic losses create temperature gradients within the GIS^32^. This suggests that SF6 gas is highly sensitive to ambient temperature variations, and frequent operations and sudden temperature changes should be minimized during operation.

**Table 1:** Leakage event log data.

Time of leakage occurrence	Leak location	Leakage severity	
2024/1/15 17:13:00		Gas Compartment	Moderate Leakage

**Table 2:** Comparative analysis of gas parameters before and after leakage events.

Time	SF6 gas density/(kg/m³)	SF6 Gas Pressure/(MPa)	Key Loc. 1 Temp./(℃)
2024/1/15 16:13	6.234693922	0.565960797	25.09485586
2024/1/15 16:33	6.178547404	0.551849008	24.47351746
2024/1/15 16:53	6.106021969	0.538109271	25.09240664
2024/1/15 17:13	5.916633422	0.533845372	25.40348071
2024/1/15 17:33	5.874724005	0.525984638	23.84172656
2024/1/15 17:53	5.923662617	0.514439886	22.75893069

SF6 gas leakage leads to a reduction in gas quantity inside the equipment, resulting in decreased density and pressure according to thermodynamic principles. The Joule-Thomson effect during gas escape causes a transient temperature drop. The data in Table 1 records the times when the leakage incidents occurred. As shown in Table 2, recorded data from a leakage event at 17:13 demonstrate the gas density decreased from 6.2 kg/m³ to 6.1 kg/m³, while the pressure dropped from 0.55 MPa to 0.54 MPa. Correspondingly, the temperature exhibited a brief decline from 25 °C to 24.8–24.9 °C following the leakage.

### Leakage current of the surge arrester at different voltage levels

Leakage current of the surge arrester is measured by a core-through current transformer, covering a range from 100µA to 100 mA, with an uncertainty of ≤ 0.5%.

**Fig. 6:**
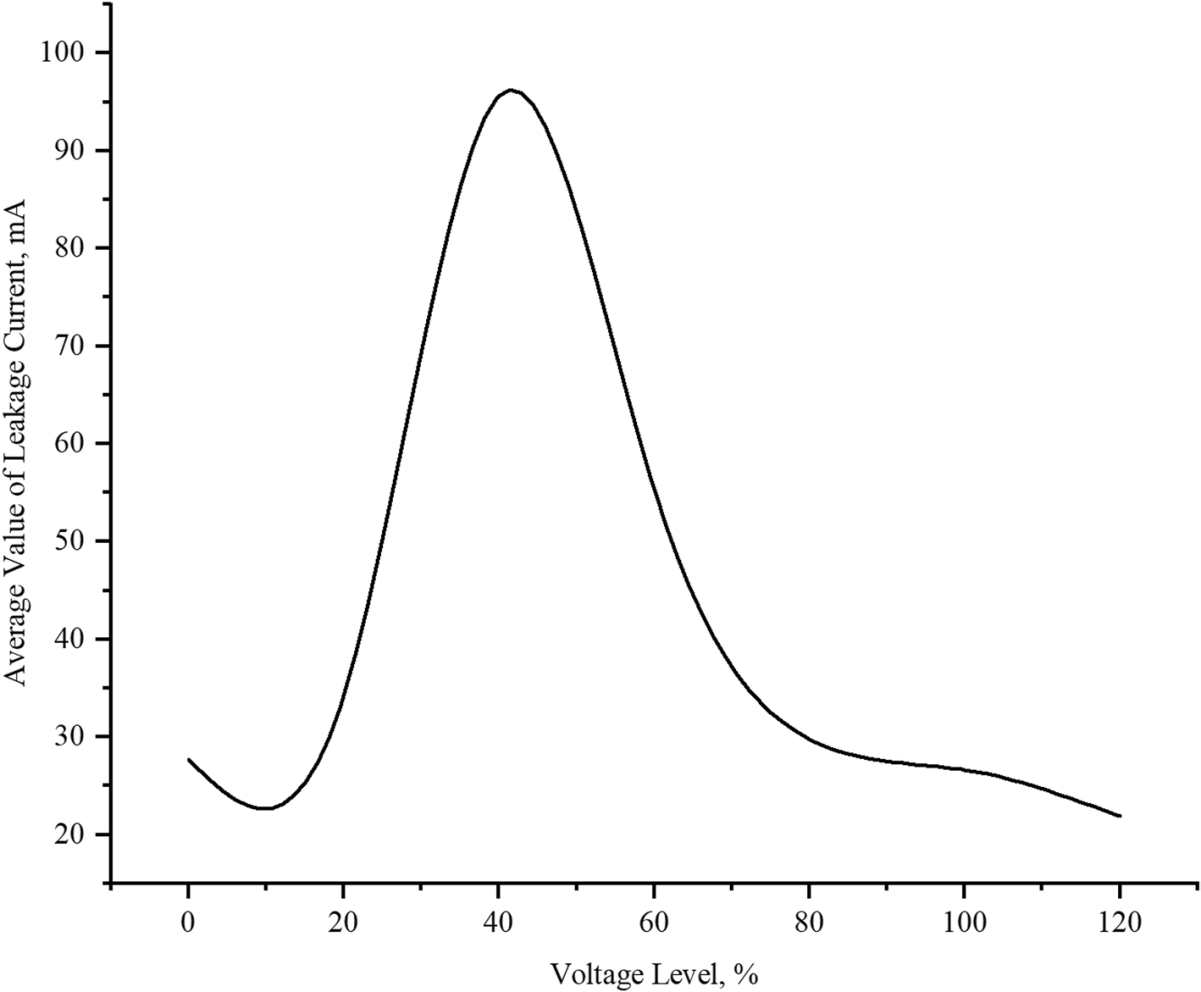
Leakage current.

Figure 6 demonstrates the nonlinear relationship between voltage levels and surge arrester leakage current. Key observations reveal: at 0% voltage, baseline leakage current measures 27.706 mA, remaining stable. A 20% voltage increase elevates current to 34.228 mA with initial fluctuations attributed to reduced air density and molecular energy changes^33^. The current peaks at 95.591 mA under 40% voltage, where thermal effects become significant - the arrester’s limited size restricts heat dissipation, causing valve disc temperature rise and consequent resistance increase due to its negative temperature coefficient. Contamination effects and microampere-scale measurements further contribute to result variations^34^. Interestingly, higher voltages (60–120%) show progressive current reduction (55.331 mA to 21.857 mA), with 100% and 120% voltages producing lower leakage than the no-load condition, indicating voltage-dependent stabilization effects. This nonlinear behavior highlights the complex interplay between voltage stress, thermal dynamics, and material properties in surge arrester performance.

**Table 3:** Time-dependent variations in SF6 gas and GIS equipment temperatures.

Time	Loc.1 SF6 Gas Temperature at/(°C)	Key Loc. 1 Temp./(℃)
2024/1/1 0:00:00	25.950595342876504	30.125224502647907
2024/1/1 1:00:00	25.44751915703806	29.281592734430802
2024/1/1 2:00:00	24.894434880586154	29.81471234112229
2024/1/1 3:00:00	25.705466189873135	31.67770081407644

Persistently elevated leakage current in surge arresters leads to increased self-heating. Since arresters are installed in close proximity to GIS equipment within the electrical system, the generated heat affects the surrounding SF6 gas temperature, potentially causing localized temperature rise in the GIS. As shown in Table 3, when arrester leakage current exceeds 25 mA, the adjacent SF6 gas temperature rises from 25 °C to 26 °C. Correspondingly, the temperature of critical GIS components increases from 30 °C to 31–32 °C. Before the voltage level reaches 40%, the leakage current increases as the applied voltage rises. This is due to the direct proportional relationship between voltage, current, and the equivalent impedance, specifically the constant capacitive impedance^35^. Since most surge arresters in use today do not have gaps, their zinc oxide discs are continuously exposed to power-frequency voltage. During operation, current always flows through the discs, and factors such as surge voltage and internal moisture can accelerate the aging of the discs, leading to an increase in resistive leakage current. Therefore, long-term online monitoring of the surge arrester is necessary to ensure its safe operation^36^.

### Number of discharge occurrences of the gas-insulated switchgear

Monitor three-phase voltage, with an accuracy of Class 0.5.

Monitor three-phase current, with an accuracy of Class 0.5.

Measure active power, with an accuracy of Class 0.5.

Measure reactive power, with an accuracy of Class 1.0.

Measure apparent power, with an accuracy of Class 0.5.

Monitor power factor, with an accuracy of Class 0.5.

The parameters were measured during Period A and Period B, respectively, with an interval of approximately half a month between the two periods.

#### Three-phase voltage under different load conditions

**Fig. 7:**
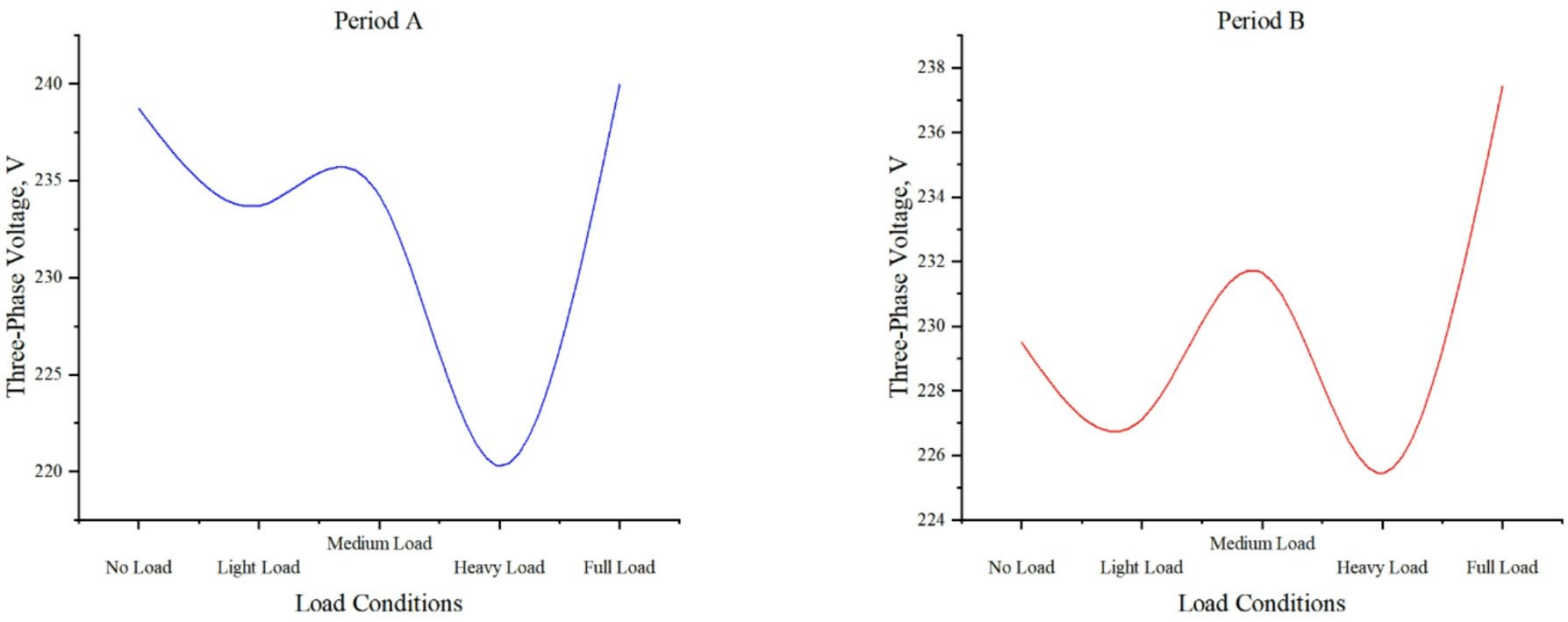
Distribution of three-phase voltage and load levels in time period A and time period B.

Figure 7 presents three-phase voltage characteristics under varying load conditions. Key observations include: no-load conditions maintain stable voltages (238.752 V, 229.505 V), while light loads (233.697 V, 227.108 V) exhibit minor fluctuations with an overall upward trend. Medium loads sustain voltages above 230 V (234.238 V, 231.648 V), indicating stable operation. Heavy loads demonstrate voltage depression (220.275 V, 225.446 V), revealing system stress. Notably, full-load conditions show rapid voltage recovery (239.958 V, 237.423 V), approaching maximum operational values. This nonlinear response highlights the system’s load-dependent voltage regulation characteristics, with significant variations occurring primarily during heavy and full-load conditions. The data suggests effective voltage maintenance across most operating ranges except under heavy load stress.

**Table 4:** GIS equipment temperature distribution.

Time	Key Loc. 1 Temp./(℃)	Key Loc. 1 Temp./(℃)	Key Loc. 1 Temp./(℃)
2024/1/1 0:00:00	30.125224502647907	29.570594458874748	30.122297503032282
2024/1/1 1:00:00	29.281592734430802	30.89492437694444	29.705050321707972
2024/1/1 2:00:00	29.81471234112229	29.870179300266575	30.043811471979108
2024/1/1 3:00:00	31.67770081407644	29.446411758292005	30.568983079185276

Voltage fluctuations in power systems significantly alter the electric field distribution within gas-insulated switchgear. When the system voltage exceeds standard levels or experiences large fluctuations, the resulting electric field non-uniformity intensifies partial discharge activity. Refer to Table 4, this phenomenon produces two critical effects: (1) localized heating, elevating temperatures in critical components from approximately 30 °C to 35 °C, and (2) accelerated aging of insulation materials due to prolonged PD exposure.

Internal defects in GIS can trigger persistent partial discharge, degrading insulation performance. This leads to minor voltage fluctuations, which further accelerate equipment deterioration. When partial discharge exceeds 50 pC, voltage fluctuations typically widen from ± 0.5 V to ± 1 V or more. This expanded fluctuation range intensifies insulation stress, creating a degradation feedback loop. One of the methods to protect transmission lines from lightning overvoltage is by installing lightning arresters^37,38^. The lightning arrester limits the peak overvoltage to a level that prevents damage to the equipment^39^. By examining these data points, curve trends, and condition labels, we can gain a deeper understanding of how operating conditions affect the discharge counts of the lightning arrester and the overall performance of the system.

#### Three-phase current under different load conditions

**Fig. 8:**
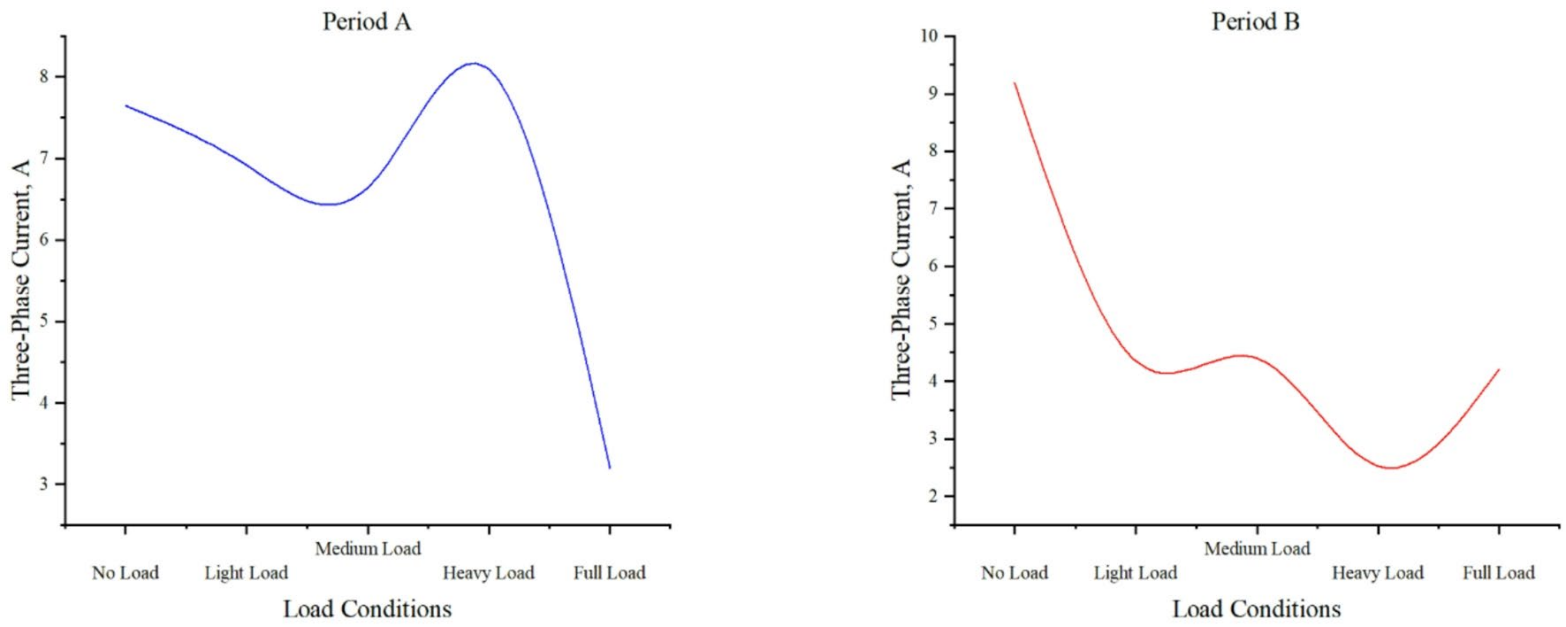
Distribution of three-phase current and load levels in time period A and time period B.

Figure 8 demonstrates three-phase current behavior under varying load conditions, revealing distinct operational patterns. The no-load condition shows stable currents (7.650 A, 9.196 A) with a slight downward trend. Light and medium loads exhibit current stabilization after initial fluctuations (light: 6.920 A, 4.355 A; medium: 6.641 A, 4.404 A), suggesting effective system regulation during moderate operation. Heavy load conditions display marked instability (8.093 A, 2.519 A), evidenced by significant current variations that highlight system stress. Full-load operation returns to stabilized currents (3.198 A, 4.215 A), indicating system adaptation to maximum demand conditions. These findings reveal critical load-current relationships, particularly the pronounced instability during heavy loading and the system’s capacity to stabilize under both minimal and full-load conditions, providing valuable insights for electrical system performance analysis and load management strategies.

#### Active power under different load conditions

**Fig. 9:**
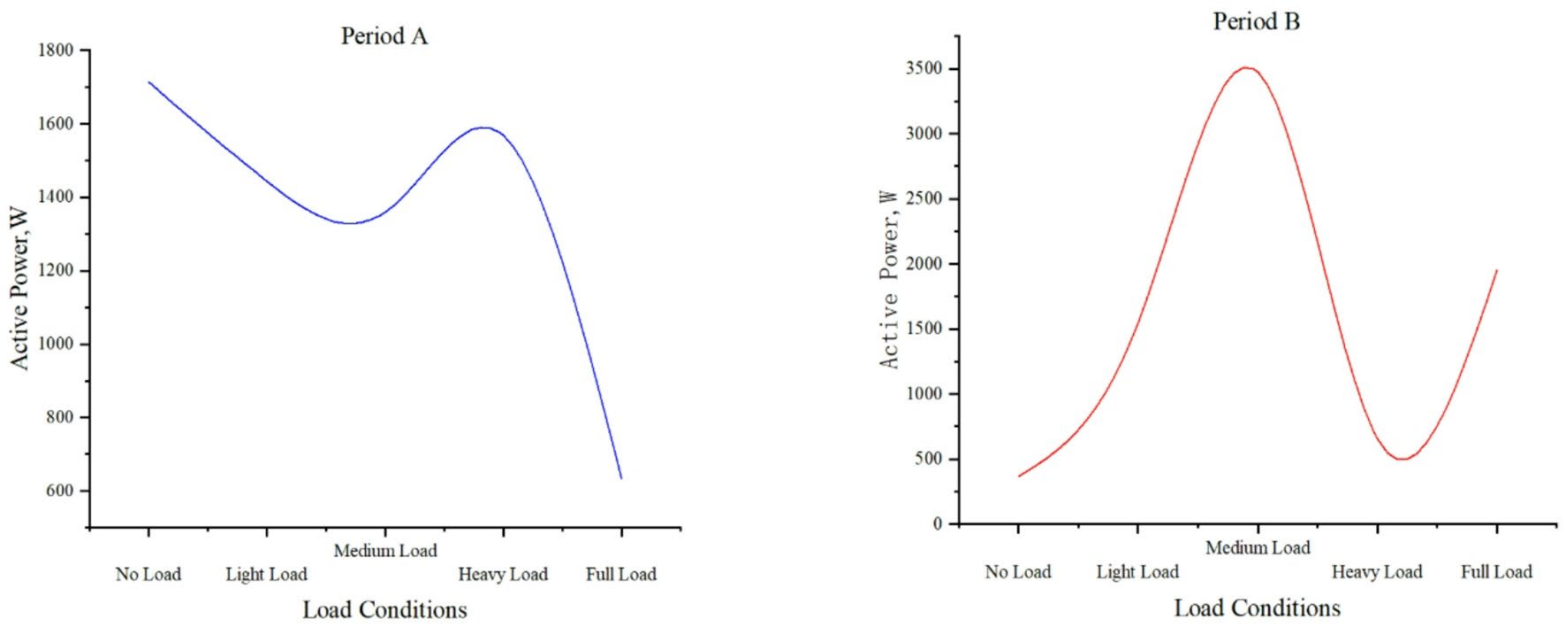
Distribution of active power and load levels in time period A and time period B.

Figure 9 demonstrates active power characteristics across load conditions, revealing distinct operational patterns. No-load shows significant variation (1713.494 W, 362.905 W), while light load maintains stability (1444.309 W, 1538.592 W). Medium load exhibits the widest range (1359.609 W, 3472.504 W) with a downward trend, contrasting with heavy load’s reduced fluctuations (1567.942 W, 651.965 W). Full load displays moderate variation (632.944 W, 1956.000 W) with stabilization tendency. The non-linear characteristics highlight medium load’s highest variability and full load’s adaptive stabilization, providing critical insights for power system optimization.

**Table 5:** Active power and leakage current at different time intervals.

Time	Active Power Load/(MW)	Average leakage current/(mA)
2024/1/1 0:00:00	93.61038252310497	20.395309462458485
2024/1/1 1:00:00	116.4201516013637	19.02362472871663
2024/1/1 2:00:00	93.11849654822277	19.15971856934287
2024/1/1 3:00:00	109.81765486991594	19.47392196778804
2024/1/1 4:00:00	75.00594285126427	18.237167516157307

Refer to Table 5 Changes in system load affect line current and voltage. A sudden rise in active power may cause voltage fluctuations or overvoltage. Surge arresters activate to protect equipment during overvoltage, with operation frequency and leakage current increasing accordingly. When active power increases, arrester operations often rise from 0 to 1 + event. Under heavy load, average leakage current increases from 17 to 20 mA (no load) to 30–35 mA.

#### Reactive power under different load conditions

**Fig. 10:**
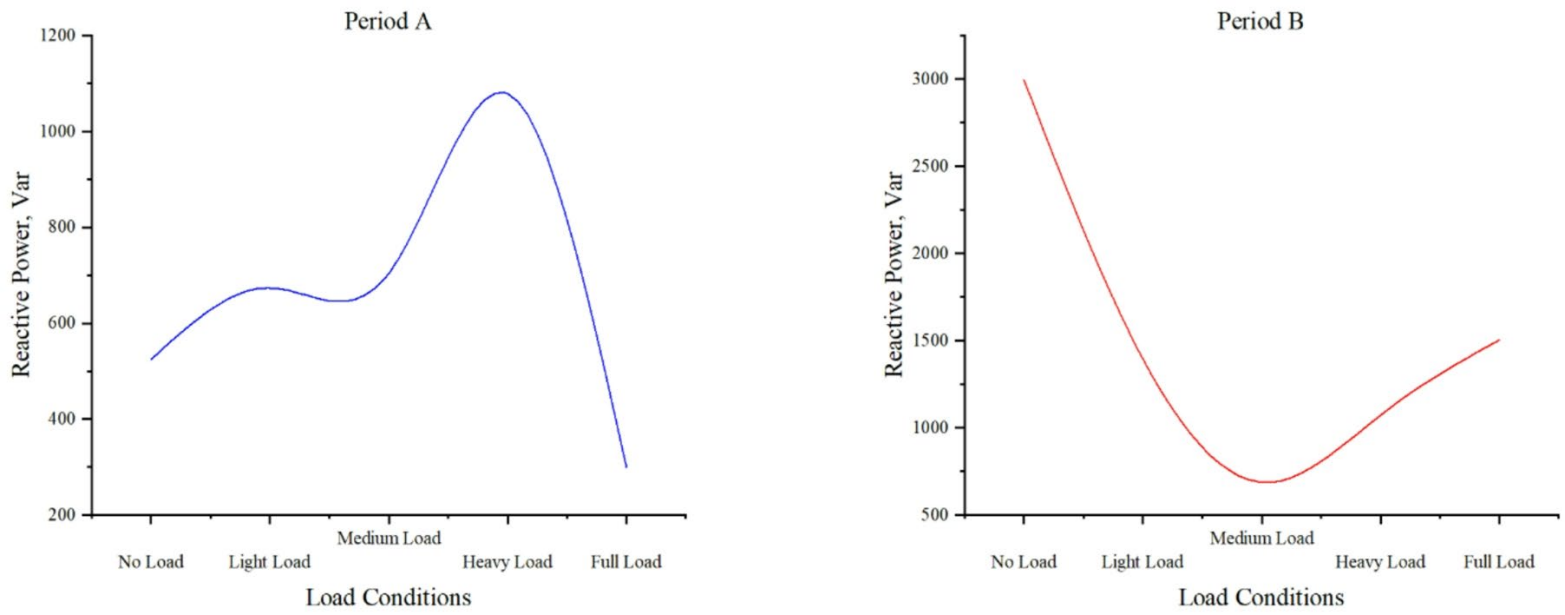
Distribution of reactive power and load levels in time period A and time period B.

Figure 10 demonstrates reactive power variations across load conditions. No-load exhibits significant variation (524.115Var, 2998.016Var), while light load shows moderate fluctuations within a defined range (674.060Var, 1397.298Var). Medium and heavy load conditions maintain relative stability (704.791Var, 687.800Var and 1078.828Var, 1074.092Var respectively). Full load displays notable instability (298.470Var, 1506.183Var), indicating a transition from stable to unstable operation. These patterns reveal load-dependent reactive power characteristics critical for system performance analysis.

#### Apparent power under different load conditions

**Fig. 11:**
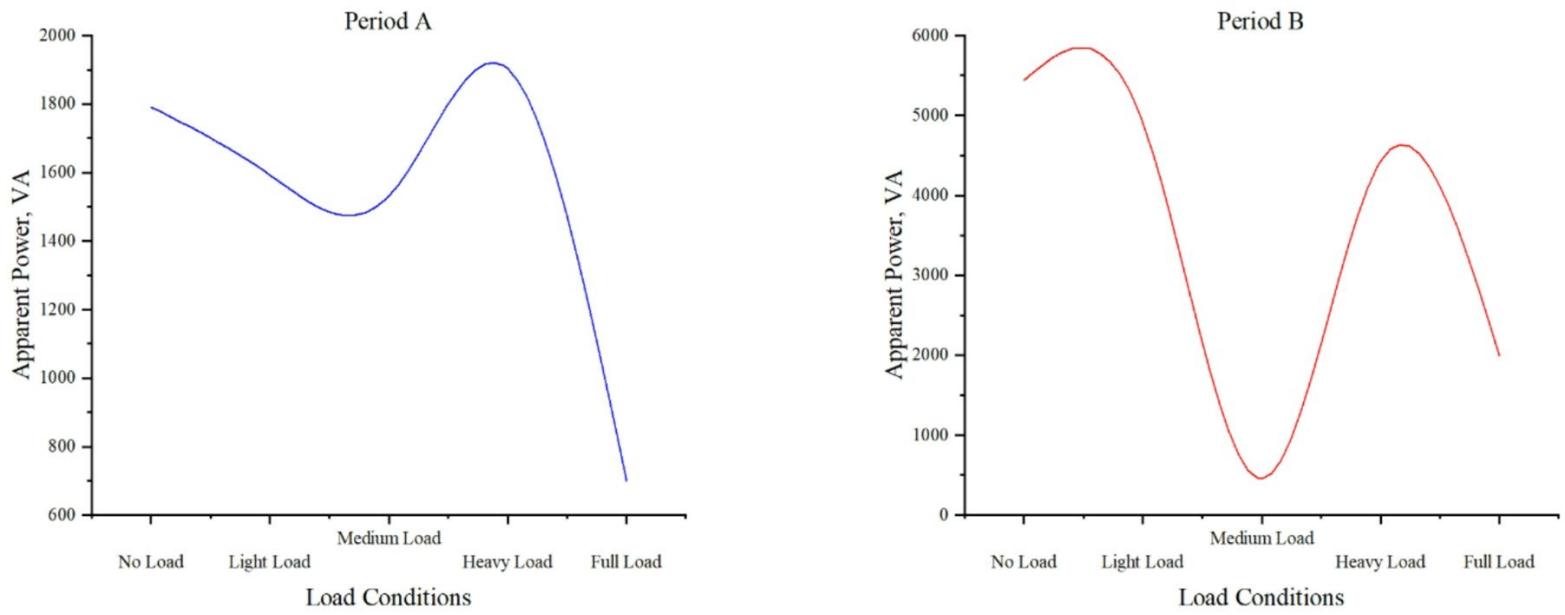
Distribution of Apparent Power and Load Levels in Time Period A and Time Period B.

Figure 11 demonstrates apparent power characteristics across load conditions. No-load shows significant variation (1791.858VA, 5441.769VA), while light load exhibits downward-trending fluctuations (1593.859VA, 4924.078VA). Medium load maintains stable operation with an upward trend (1531.426VA, 450.743VA), and heavy load shows consistent values (1903.237VA, 4431.724VA). Full load displays considerable fluctuations at reduced levels (699.787VA, 1989.346VA). These patterns reveal distinct load-dependent behaviors, with medium and heavy loads demonstrating optimal stability compared to more variable no-load and full-load conditions. The data provides critical insights for system performance evaluation under varying electrical loads.

#### Power factor under different load conditions

**Fig. 12:**
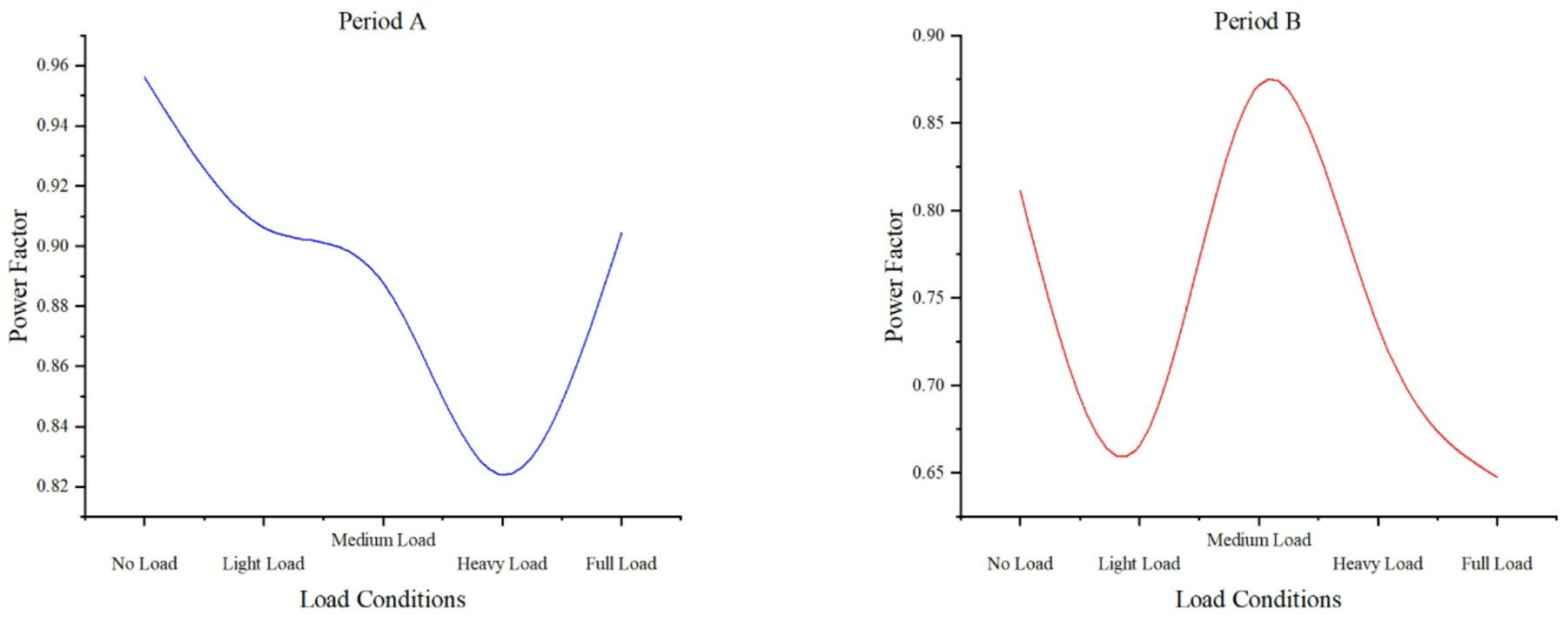
Distribution of power factor and load levels in time period A and time period B.

Figure 12 demonstrates power factor variations across load conditions. No-load maintains stable values (0.956, 0.811), while light load shows a downward trend (0.906, 0.666). Medium load achieves stable, high performance (0.888, 0.872), contrasting with heavy load’s moderate stability (0.824, 0.733). Full load exhibits significant instability (0.904, 0.648), with reactive power fluctuations being the primary contributor. Environmental factors, particularly moisture affecting lightning arresters, further influence measurements by increasing resistive current without altering capacitive current^40^. The data reveals optimal power factor stability at medium loads, with increasing variability under both lighter and full-load conditions, highlighting load-dependent electrical characteristics critical for system performance analysis.

The GIS monitoring data remains unstable, suggesting that it is highly sensitive to external factors such as temperature and humidity^41^. The overall operational parameters of GIS fluctuate to varying extents, and except for the three-phase voltage, data measured at different times exhibit significant variations. As a result, operational parameters from different time periods were selected for comparative analysis. Comparative analysis shows that as the load increases from light to heavy load conditions, the measured data exhibit a trend from stabilization to stability, followed by instability. This suggests that increasing the load on the GIS during normal operation leads to significant fluctuations in its parameters. Therefore, it is crucial to avoid overload conditions to ensure the equipment operates within the normal load range.

## Correlation analysis and equipment condition assessment

### Monitoring ultra-high voltage electrical equipment condition monitoring system design

**Fig. 13:**
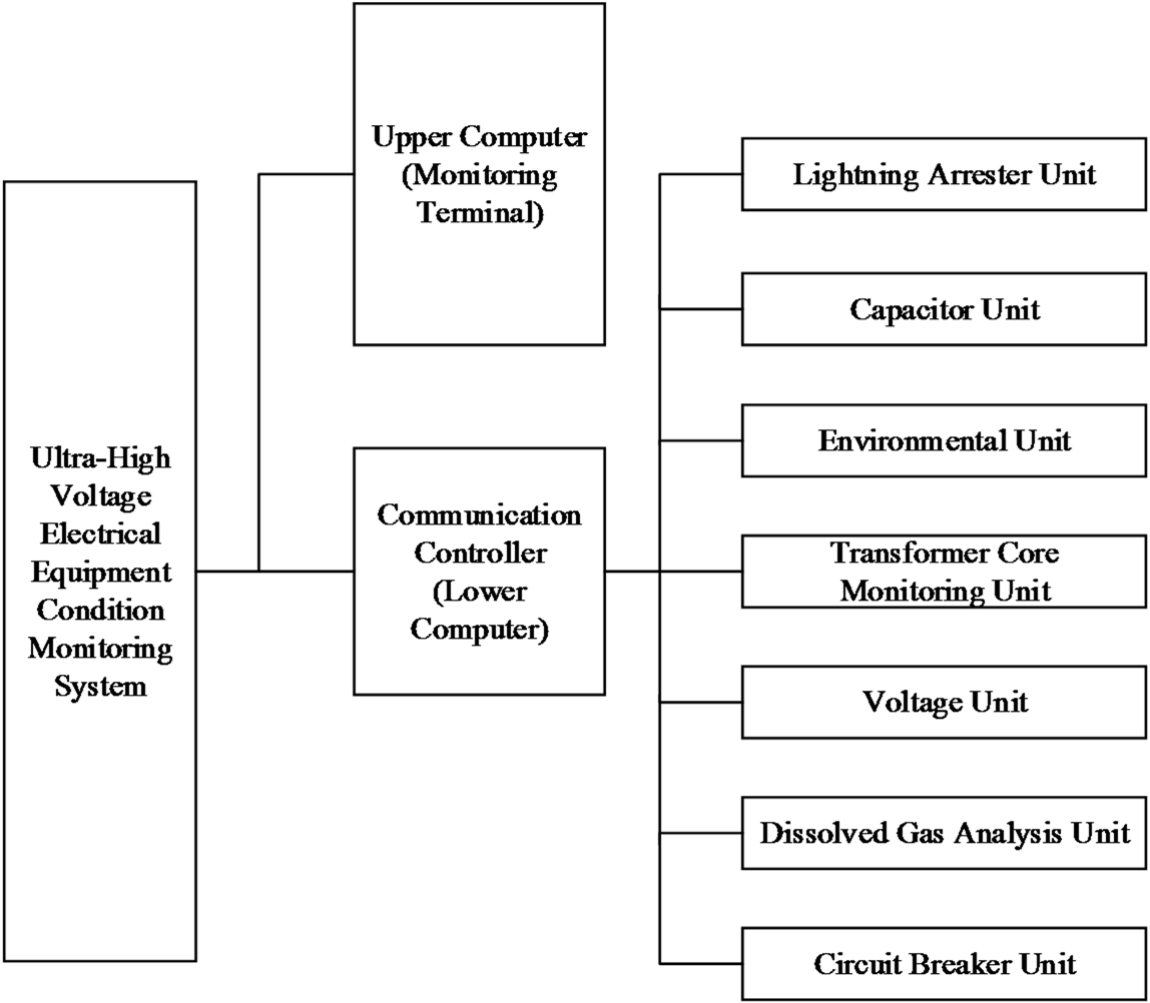
Block Diagram of the Ultra-High Voltage Electrical Equipment Condition Monitoring System Design.

The architecture of the Ultra-High Voltage Electrical equipment monitoring system, as illustrated in Fig. 13, adopts a hierarchical distributed structure comprising three functional layers: (1) Local Measurement Layer - Device-specific sensors (e.g., for arresters, transformers, CT/PT) perform on-site data acquisition and signal conditioning; (2) Communication Control Layer - Utilizing RS-485 networks within substations, this layer handles signal preprocessing, A/D conversion, and synchronized data collection across all monitoring units, while facilitating data transmission to the host computer; (3) Diagnostic System Layer - Advanced analytics process real-time condition monitoring data using intelligent algorithms to evaluate equipment health, generating actionable insights for maintenance planning and grid stability assurance.

This study adopts a hybrid intelligent algorithm architecture combining supervised learning (Random Forest for normal/warning/fault classification), LSTM networks for analysing SF₆ gas and leakage current trends, and physics-based reasoning for cross-validation. For instance, the on-site monitoring unit for arresters collects leakage current from each phase. The data is transmitted via bus to the data acquisition and control unit, where it is aggregated and processed before being uploaded to the backend online monitoring and fault diagnosis system in the central control room. This system utilizes mathematical morphology, fuzzy neural networks, and expert systems to perform online monitoring and fault diagnosis for the arresters. The three-layer system (data acquisition, feature extraction, and diagnosis) maintains stable performance under ± 10% voltage fluctuations and − 20 ~ 50℃ temperatures. By implementing dynamic threshold adjustment and multi-source data fusion, it significantly reduces false alarms compared to conventional systems, though sensor data quality (e.g., dust interference with infrared measurements) remains a limitation for future optimization.

### Core analytical dimensions for data correlation in GIS condition monitoring

#### Temporal correlation analysis

Temporal correlation analysis of synchronized parameter variations reveals causal relationships in power equipment. System voltage fluctuations trigger measurable changes in GIS partial discharge activity and temperature within minutes, while SF6 leakage events induce progressive alterations in gas density, pressure, and temperature profiles over subsequent time intervals, establishing definitive cause-effect sequences.

#### Multi-parameter cross-validation

Integrated analysis of multiple parameters enables more accurate assessment of equipment operating conditions and potential faults. When system frequency deviation exceeds thresholds, GIS partial discharge activity increases, and SF6 gas density decreases simultaneously, the probability of equipment failure rises significantly. Cross-verification of these parameters facilitates early detection of latent faults, allowing timely preventive measures to be implemented.

### Data-driven equipment condition assessment logic

#### Normal operation

The system maintains stable voltage at 220 ± 2 V and frequency at 50 ± 0.02 Hz. The GIS equipment temperature remains below 35 °C with zero partial discharge. The leakage current of surge arresters is less than 20 mA. The SF6 gas density stays within 6.2 ± 0.1 kg/m³, with pressure maintained at 0.55 ± 0.01 MPa.

#### Early warning

The system exhibits voltage fluctuations within ± 5 V and frequency deviations up to ± 0.1 Hz. GIS equipment temperatures range between 35 and 40 °C with partial discharges of 10–50 pC. Surge arresters show leakage currents of 20–25 mA, while SF6 gas density varies between 6.0 and 6.1 kg/m³ (indicating potential minor leakage).

#### Fault condition

The system demonstrates voltage fluctuations exceeding ± 10 V and frequency deviations beyond ± 0.5 Hz. GIS equipment temperatures surpass 40 °C with partial discharges > 100 pC. Surge arresters exhibit leakage currents > 25 mA with at least one operation recorded. SF6 gas density falls below 5.5 kg/m³ (indicating moderate-to-severe leakage).

### Factors affecting the accuracy of online monitoring systems

The online monitoring system utilizes voltage signals from bus potential transformer (PT) secondary terminals as reference measurements. However, inherent PT characteristics and secondary load effects introduce phase angle deviations, leading to inaccuracies in loss angle measurements. Practical operational factors—including voltage fluctuations, frequency variations, and load changes—further contribute to phase angle drift. To mitigate harmonic interference inherent to power grids, the system incorporates compensation circuits and harmonic filters in its signal processing unit, effectively reducing voltage distortion effects on measurement precision^42^. Additionally, surge arresters in substations exhibit electrical coupling through inter-phase distributed capacitance, causing their operating states to be jointly influenced by inter-phase capacitance and self-voltage. The system addresses this interference by deploying compensation capacitors and implementing advanced signal processing techniques to correct dielectric loss angle measurement errors.

### Comparative cost analysis between this system and traditional monitoring systems

**Table 6:** Cost composition of traditional monitoring systems.

Component	Unit Cost (¥)	Quantity	Annual Cost (¥)	Remarks
Manual Inspection (2 technicians × 2 rounds/month)	800/round	48	38,400	High-voltage equipment access required (safety hazards)
SF6 Gas Pressure Monitor	1,500	3	4500	Limited accuracy, requires periodic replacement
Infrared Thermography Camera (Handheld)	25,000	1	5,000 (depreciated)	5-year operational lifespan
Ground Leakage Detector	3000	2	6000	Annual calibration mandatory
Estimated Annual Losses (False Alarms/Undetected Faults)	N/A	N/A	10,000	System outages due to delayed fault recognition
Total			63,900	

**Table 7:** Cost structure of the intelligent online monitoring system.

Item	Unit Cost (¥)	Quantity	Initial Investment/Annual Amortization (¥)	Remarks
Infrared Sensing Module	2,500	3	7,500/5 ≈ 1,500	Service life ≥ 5 years
SF6 Gas Density/Pressure/Temperature Integrated Sensor	1,500	3	11,400/5 ≈ 2,280	Digital output, high accuracy
Leakage Current Monitoring Module (with wireless upload)	25,000	2	5,200/5 ≈ 1,040	Supports alarm linkage
Data Acquisition Terminal + Processing Module	3000	1	5,000/5 ≈ 1,000	Multi-channel acquisition
Wireless Communication & Cloud Platform Service	-	-	2000	Includes data storage and visualization
Annual Maintenance (calibration & system maintenance)	-	-	3000	Includes software upgrades
False Alarm Avoidance (power outage loss prevention)	-	-	0	High-reliability fault identification
Total			10,820	

**Table 8:** Comparison analysis summary.

Comparison Item	Traditional System	This System	Cost Advantage
Annual Total Cost	≈¥63,900	≈¥10,820	Over 82% savings
Alarm Accuracy	High false alarm rate (up to 100%)	Low false alarms with integrated analysis	Data fusion & trend prediction
Real-time Performance & Continuity	Long inspection intervals	Real-time collection, second-level upload	Timely fault warnings
Human Resource Dependency	High	Minimal (automatic operation)	Reduces O&M staffing needs
Service Life	Equipment < 5 years	Equipment lifespan ≥ 5 years	Shorter ROI period
Safety	Risks from manual HV equipment access	Remote online monitoring	Eliminates tower climbing

As ultra-high voltage electrical equipment becomes more complex, condition monitoring must be more accurate and real-time. Table 6 presents the cost structure of conventional monitoring systems, while Table 7 details the cost composition of our proposed system. A comprehensive comparative cost analysis between traditional and our systems is summarized in Table 8. Unlike manual inspections or basic sensor systems, our intelligent online monitoring system improves detection accuracy, reduces false alarms, and extends equipment life. It also offers better long-term cost savings than traditional systems.

## Analysis of false alarm incidents in surge arrester online monitoring systems

Current online monitoring systems for electrical equipment face significant challenges, including inconsistent device performance and technical limitations that compromise data reliability^43^. As evidenced by a 2015 case study from Fujian Electric Power Company involving 247 surge arrester monitoring devices across 14 substations, these systems demonstrated 100% false alarm rates (73 false alerts with zero valid alarms or defect detections). Analysis revealed that 79.4% of false alarms stemmed from erroneous resistive current measurements, while 20.6% originated from total leakage current inaccuracies^44^. To address these issues, our system implements multi-parameter correlation analysis and cross-validation, effectively mitigating false alerts caused by environmental interference, hardware failures, and software errors through comprehensive data verification.

## Conclusion

This paper provides an accurate assessment of the operational status of electrical equipment by monitoring operational data and utilizing analytical methods, allowing for early warning of potential failures. Detailed data monitoring and analysis are carried out in four key areas of the GIS system: gas parameters, leakage current, discharge counts, and electrical parameters.

1. The GIS’s SF6 gas exhibits significant operational variations: density drops to 5.529 kg/m³ during switching but recovers to 6.452 kg/m³ with temperature rise, demonstrating clear temperature sensitivity; SF₆ gas pressure is 0.494 MPa under normal operation, and rises under other operating conditions. No low-pressure alarms were triggered during testing, compared to the actual alarm threshold of 0.42 MPa; temperature ranges from − 28.491 °C (frequent switching) to 36.468 °C (normal), strongly suggesting the need to minimize frequent switching operations.
2. As the voltage level rises, the leakage current of the lightning arrester reaches 95.591 mA at around 40% of the voltage level, then starts to decrease. When the voltage level reaches 120%, the leakage current drops to 21.857 mA. The waveform stabilizes as the voltage level nears 100%.
3. The three-phase voltage ranges from 220.275 V (heavy load) to 239.958 V (full load). Current drops to 2.519 A under heavy load with 5.574 A peak-to-peak fluctuation, showing an overall decline. Active power peaks at 3,472.504 W under medium load then stabilizes. Reactive power reaches 1,078.828 var under heavy load with 1,207.713 var variation. Apparent power hits 4,431.743 VA under heavy load, waveform inflecting at medium load. Power factor dips to 0.733 under heavy load, directly load-dependent.

This paper presents insights into the condition of electrical equipment and establishes a condition monitoring system, which helps improve equipment reliability, and reduce operation and maintenance costs. The fault warning system promptly issues alerts for anomalies, which helps to prevent equipment failures.

## Supplementary Information

Below is the link to the electronic supplementary material.

Supplementary Material 1

## Data Availability

The datasets used and/or analysed during the current study are available from the corresponding author on reasonable request.
